# Prognostic Value of the C-reactive Protein-to-Lymphocyte Ratio (CLR) for Lupus Nephritis: Comparison With Neutrophil-to-Lymphocyte Ratio (NLR) and Fibrinogen-to-Albumin Ratio (FAR)

**DOI:** 10.7759/cureus.92915

**Published:** 2025-09-22

**Authors:** Lijuan Zhao, Feng Chen, Jie Hong, Chuyu Shen, Yuxuan Zhang, Xianming Zhang, Ming Gui

**Affiliations:** 1 Department of Rheumatology, Third Xiangya Hospital of Central South University, Changsha, CHN; 2 Department of Preventive Health Care, Second People's Hospital of Guizhou Province, Guiyang, CHN; 3 Department of Nephrology, Third Hospital of Changsha, Changsha, CHN; 4 Department of Critical Care Medicine, Third Xiangya Hospital of Central South University, Changsha, CHN

**Keywords:** c-reactive protein-to-lymphocyte ratio (clr), fibrinogen-to-albumin ratio (far), lupus nephritis, neutrophil-to-lymphocyte ratio (nlr), prognosis

## Abstract

Background

This study aimed to evaluate the prognostic value of C-reactive protein-to-lymphocyte ratio (CLR) in lupus nephritis (LN), and compare its utility with neutrophil-to-lymphocyte ratio (NLR) and fibrinogen-to-albumin ratio (FAR).

Methods

We retrospectively enrolled 359 newly diagnosed or immunosuppressant-free LN patients, including 215 with renal biopsy data. A composite endpoint was used to define poor renal prognosis. Baseline CLR, NLR, and FAR at first hospitalization were compared between patients with and without poor prognosis, and their correlations with clinical characteristics were analyzed. Prognostic value was assessed using receiver operating characteristic (ROC) analysis, Kaplan-Meier survival analysis, and least absolute shrinkage and selection operator (LASSO) Cox regression.

Results

Over a median follow-up of 36 months, 137 patients reached the composite endpoint. Baseline CLR, NLR, and FAR were significantly elevated in the endpoint group. CLR exhibited the strongest correlation with disease activity (SLEDAI) compared to NLR and FAR, and correlated significantly with active pathological lesions (activity index, endocapillary hypercellularity). The area under the ROC curve (AUC) for CLR in predicting poor prognosis was 85.94%, significantly higher than that of NLR or FAR. Patients with CLR >2.23 (optimal cut-off) had significantly poorer renal outcomes. LASSO Cox regression identified CLR as an independent predictor of poor renal prognosis, regardless of whether pathological parameters were included.

Conclusion

CLR demonstrates superior prognostic value for renal outcomes in LN compared to NLR and FAR. Its strong association with disease activity and pathological findings suggests CLR may serve as a valuable, readily available early warning biomarker in clinical practice.

## Introduction

Systemic lupus erythematosus (SLE) is a complex autoimmune disease characterized by inflammation and immune-mediated injury to multiple organ systems. Up to 50% SLE patients develop lupus nephritis (LN) during their disease course, with a high risk of progression to end-stage renal disease (ESRD) requiring kidney replacement therapy [1]. Early identification of patients at high risk for poor renal outcomes remains a critical challenge in LN management.

Renal biopsy is pivotal for assessing disease activity and guiding treatment, as emphasized in major LN management guidelines. However, as an invasive procedure carrying risks such as hemorrhage and infection, its routine application is limited. Consequently, there is an urgent need for novel, readily accessible biomarkers with robust prognostic capabilities.

The C-reactive protein (CRP)-to-lymphocyte ratio (CLR) is an emerging inflammation-based biomarker that has demonstrated prognostic value in various conditions, including malignancies, COVID-19, and acute pancreatitis [2-4]. Despite its promise, the role of CLR in predicting renal prognosis in LN has not been investigated.

Accumulating evidence also suggests that other composite inflammatory indices derived from routine blood tests, such as the neutrophil-to-lymphocyte ratio (NLR) and fibrinogen-to-albumin ratio (FAR), correlate with SLE disease activity and renal involvement [5-7]. These markers are cost-effective and easily accessible, rendering them promising candidates for clinical use. Nevertheless, the comparative prognostic utility of these indices in LN remains inadequately explored. Therefore, this study aimed to evaluate the prognostic value of baseline CLR, measured at the time of first hospitalization, for renal outcomes in LN, and to compare its performance with established indices, including NLR and FAR.

## Materials and methods

Patients and study design

This retrospective longitudinal study included LN patients admitted to the Third Xiangya Hospital of Central South University between January 2012 and December 2023. The inclusion and exclusion criteria were summarized in Table 1. SLE was diagnosed according to the 1997 American College of Rheumatology (ACR) classification criteria, which requires the presence of at least four out of the following 11 clinical and immunologic criteria: malar rash, discoid rash, photosensitivity, oral ulcers, non-erosive arthritis, serositis (pleuritis or pericarditis), renal disorder (proteinuria or cellular casts), neurologic disorder (seizures or psychosis), hematologic disorder (hemolytic anemia, leukopenia, lymphopenia, or thrombocytopenia), immunologic disorder (anti-dsDNA, anti-Sm, or antiphospholipid antibodies), and a positive antinuclear antibody (ANA) test. LN required SLE to fulfill at least one of the following renal criteria [8]: 1) persistent proteinuria >0.5g/24h or spot urine protein-creatinine ratio >500mg/g (50mg/mmol); 2) active urinary sediment (>5 red blood cells (RBCs)/high-power field (HPF), >5 white blood cells (WBCs)/HPF, or cellular casts in the absence of infection); or 3) renal biopsy demonstrating immune complex-mediated glomerulonephritis compatible with LN. Exclusion criteria were: 1) age <18 years; 2) concomitant non-LN nephropathy; 3) end-stage renal disease (ESRD), defined as requiring dialysis, kidney transplantation, or an estimated glomerular filtration rate (eGFR) <15 ml/min/1.73m²; 4) patients who had received immunosuppressive therapy within the preceding six months (to minimize the potential confounding effects of recent immunomodulation on baseline inflammatory indices); 5) active infections, heart disease, primary hematologic disease, or cancer; 6) extensive missing data; or 7) follow-up duration <12 months.

**Table 1:** Inclusion and exclusion criteria of the study ACR - American College of Rheumatology; SLE - systemic lupus erythematosus; RBC - red blood cell; HPF - high-power field; WBC - white blood cell; LN - lupus nephritis; ESRD - end-stage renal disease; eGFR - estimated glomerular filtration rate

Inclusion criteria	Exclusion criteria
1. 1997 ACR criteria for SLE	1. Age <18 years
2. Criteria for renal involvement (fulfilling at least one of the following)	2. Concomitant non-LN nephropathy
2.1 Persistent proteinuria >0.5g/24h or spot urine protein-creatinine ratio >500mg/g (50mg/mmol)	3. ESRD, defined as requiring dialysis, kidney transplantation, or eGFR <15 ml/min/1.73m²
2.2 Active urinary sediment (>5 RBCs/HPF, >5WBCs/HPF, or cellular casts in the absence of infection)	4. Immunosuppressive therapy within the preceding 6 months
2.3 Renal biopsy demonstrating immune complex-mediated glomerulonephritis compatible with LN	5. Active infections, heart disease, primary hematologic disease, or cancer
	6. Extensive missing data
	7. Follow-up duration <12 months

Patients were followed until reaching a composite endpoint defining poor renal prognosis. This endpoint comprised any one of the following [9,10]: 1) ≥50% reduction in eGFR from baseline; 2) a doubling of serum creatinine (Scr) levels; 3) progression to ESRD (eGFR <15 ml/min/1.73 m2); 4) initiation of dialysis, renal transplantation, or death.

**Figure 1:**
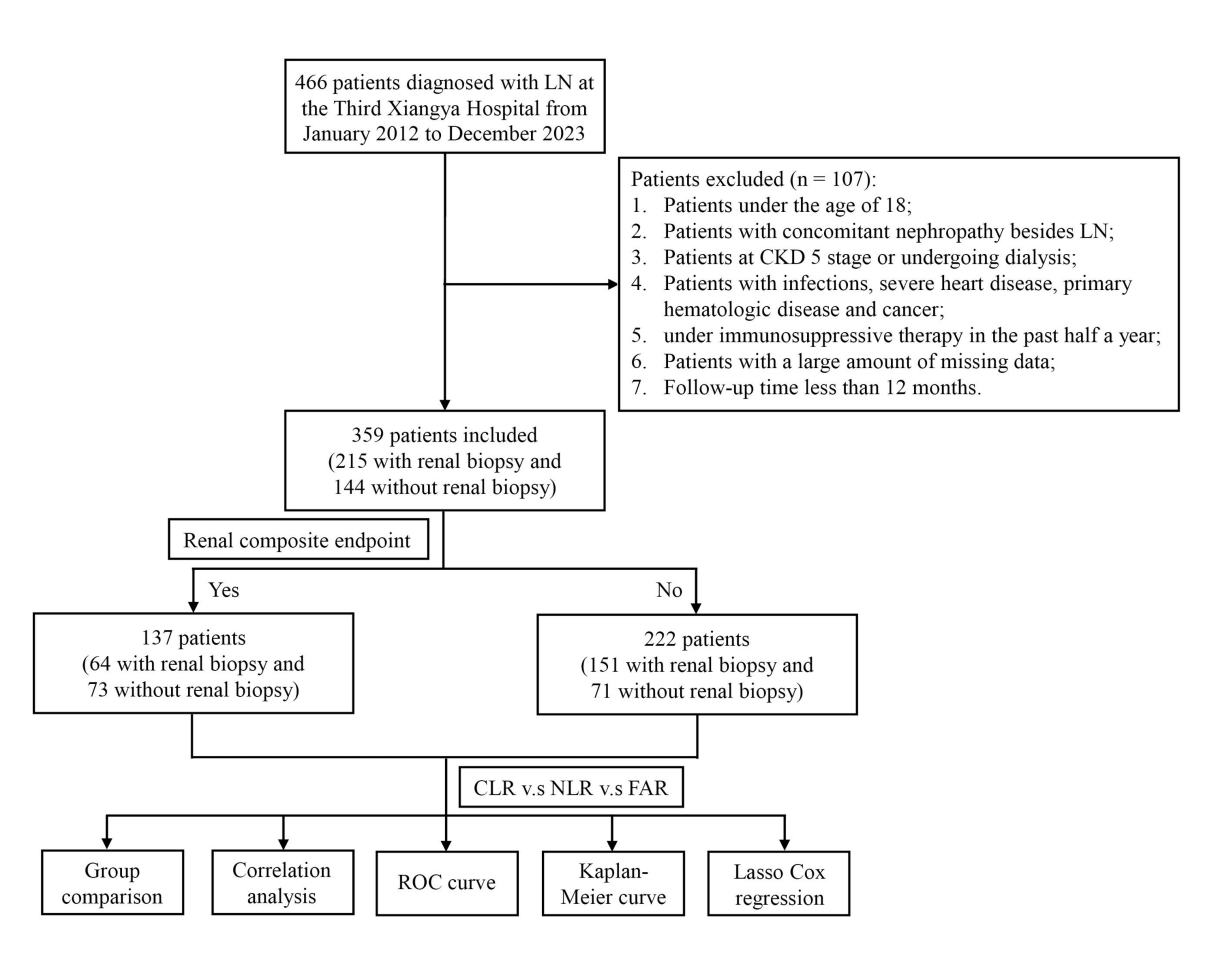
Flow diagram of the study LN - lupus nephritis; ESRD - end-stage renal disease; CLR - C-reactive protein-to-lymphocyte ratio; NLR - neutrophil-to-lymphocyte ratio; FAR - fibrinogen-to-albumin ratio; ROC - receiver operating characteristic; LASSO - least absolute shrinkage and selection operator

The study adhered to the principles of the Declaration of Helsinki and was approved by the Ethics Committee/Institutional Review Board of Third Xiangya Hospital. Written informed consent was waived due to the retrospective nature of the study. The study protocol is presented in Figure 1.

Data collection

Baseline demographic, clinical, laboratory, and pathological data were collected from the first hospitalization record for all included LN patients. Data on whether the composite endpoint was reached during follow-up were also recorded. Disease activity was assessed using the Systemic Lupus Erythematosus Disease Activity Index (SLEDAI) and categorized as: 0-4 (none), 5-9 (mild), 10-14 (moderate), or ≥15 (severe). The eGFR was calculated using the Chronic Kidney Disease Epidemiology Collaboration (CKD-EPI) formula. For patients undergoing renal biopsy, pathological evaluation followed the International Society of Nephrology/Renal Pathology Society (ISN/RPS) classification guidelines

Statistical analysis

Statistical analysis was performed using SPSS (version 26.0; IBM Inc., Armonk, New York) and R software (version 4.3.1; R Foundation, Vienna, Austria). A two-sided p-value <0.05 was considered statistically significant. Categorical variables are presented as counts and percentages (n, %) and were compared using the χ² test or Fisher's exact test, as appropriate. Continuous variables are expressed as mean ± standard deviation (SD) or median (interquartile range (IQR)), and compared using Student's t-test or the Mann-Whitney U test, depending on data distribution. To mitigate potential collinearity and overfitting, the least absolute shrinkage and selection operator (LASSO) Cox regression method was employed using the 'glmnet' and 'survival' packages in R. This identified independent predictors of poor prognosis. The optimal regularization parameter λ (yielding the minimum mean squared error) was selected via 10-fold cross-validation. Spearman's rank correlation coefficient was used to assess correlations between CLR, NLR, FAR, and clinical characteristics. The predictive performance of baseline CLR, NLR, and FAR for poor renal prognosis was evaluated using receiver operating characteristic (ROC) curve analysis. Cumulative renal survival probabilities were compared using Kaplan-Meier analysis with the log-rank test.

## Results

Comparison of baseline clinical characteristics, including renal pathological parameters, collected at first hospitalization in LN patients grouped by different prognoses.

A total of 359 LN patients with a median age of 36 years were enrolled according to the inclusion and exclusion criteria. Among these patients, 137 reached the composite endpoint, while 222 did not. As shown in Table 2, patients in the endpoint group exhibited a higher median SLEDAI score (p<0.001). The endpoint group had a larger proportion of renal hypertension. Furthermore, patients in the endpoint group had lower levels of lymphocytes and hemoglobin (HGB), along with elevated blood urea nitrogen (BUN), serum creatinine (Scr), uric acid (UA), cystatin C, CRP, blood β2-macroglobulin (MG), and urine α1-MG (all p<0.001). The composite inflammatory indices, CLR, NLR, and FAR, were significantly elevated in the endpoint group (p<0.001, p<0.001, and p=0.005, respectively). Among 215 patients who underwent renal biopsy, 64 patients were in the endpoint group and 151 in the non-endpoint group. Comparatively, a higher number of patients in the endpoint group were pathologically categorized as type III, IV, or III/IV ± V, collectively referred to as proliferative LN (p=0.099). The active index (AI) score and chronic index (CI) score were elevated in the endpoint group (p=0.093 and 0.004, respectively). Moreover, increased levels of C3 deposition (p=0.001) were found in the endpoint group.

**Table 2:** Baseline clinical characteristics in LN patients with different prognosis ^&^: chi-square (x2) test, degrees of freedom (df) =1, effect size = Cramer’s V (V); SLEDAI - Systemic Lupus Erythematosus Disease Activity Index; WBC - white blood cell; HGB - hemoglobin; PLT - platelet; ALB - albumin; GLB - globulin; BUN - blood urea nitrogen; SCr - serum creatinine; UA - uric acid; MG - macroglobulin; ESR - erythrocyte sedimentation rate; CRP - C-reactive protein; C3 - complement 3; C3 - complement 4; AnuA - anti-nucleosome antibodies; AHA - anti-histone antibodies; Anti-rRNP - anti-ribosomal P-protein autoantibody; NLR - neutrophil-to-lymphocyte ratio; FAR - fibrinogen-to-albumin ratio; CLR - C-reactive protein-to-lymphocyte ratio; NAG enzyme - N-Acetyl-/β-glucosaminidase enzyme; UTP - urine total protein; AI - active index; CI - chronic index; C1q - complement 1q

Variables	Total n=359	Renal composite endpoint		Z	x^2^ (V)^&^	p-value
		Yes (n=137)	No (n=222)			
Age (years)	36.0 (27.0, 46.0)	39.0 (26.5, 48.0)	35.0 (27.8, 45.0)	-1.234		0.217
Male, n (%)	49 (13.6)	27 (19.7)	22 (9.9)		6.901 (0.139)	0.009
Clinical manifestations, n (%)						
Erythra	89 (24.8)	34 (24.8)	55 (24.8)		<0.001 (<0.001)	0.993
Alopecia	27 (7.5)	12 (8.8)	15 (6.8)		0.488 (0.037)	0.485
Mucosal ulcer	15 (4.2)	8 (5.8)	7 (3.2)		1.527 (0.065)	0.217
Arthritis	79 (22.0)	23 (16.8)	56 (25.2)		3.514 (-0.099)	0.061
Serositis	50 (13.9)	24 (17.5)	26 (11.7)		2.383 (0.081)	0.123
Cardiac involvement	22 (6.1)	14 (10.2)	8 (3.6)		6.445 (0.134)	0.011
Pulmonary involvement	53 (14.8)	23 (16.8)	30 (13.5)		0.722 (0.045)	0.395
Gastrointestinal involvement	19 (5.3)	9 (6.6)	10 (4.5)		0.721 (0.045)	0.396
Neuropsychiatric involvement	25 (7.0)	13 (9.5)	12 (5.4)		2.181 (0.078)	0.14
Hematological involvement	102 (28.4)	50 (36.5)	52 (23.4)		7.118 (0.141)	0.008
Renal hypertension	110 (30.6)	57(41.6)	53(23.9)		12.534 (0.187)	<0.001
SLEDAI	15.0 (12.0, 18.0)	16.0 (14.0, 20.0)	14.0 (10.0, 16.0)	-4.964		<0.001
Blood parameters						
WBC (×10^9/L)	6.12 (4.47, 8.01)	6.18 (4.62, 8.21)	6.04 (4.47, 7.89)	-0.491		0.623
Neutrophils (×10^9/L)	4.25 (3.03, 6.03)	4.60 (3.18, 6.23)	4.08 (2.95, 5.83)	-1.568		0.117
Lymphocytes (×10^9/L)	1.09 (0.78, 1.60)	0.95 (0.66, 1.40)	1.20 (0.84, 1.82)	-4.211		<0.001
HGB (g/L)	103.0 (87.0,120.0)	94.0 (80.5, 112.0)	110.5 (97.0, 124.0)	-5.991		<0.001
PLT (×10^9/L)	179.0 (137.0,236.0)	173.0 (128.5, 224.5)	186.0 (149.5, 246.8)	-2.507		0.012
ALB (g/L)	27.9 (22.9, 32.6)	24.3 (20.3, 31.5)	28.6 (23.0, 33.1)	-1.975		0.048
GLB (g/L)	27.0 (22.7, 32.9)	27.3(22.7,34.0)	27.4 (21.7, 32.4)	-0.797		0.425
BUN (mmol/L)	5.57 (4.11, 8.37)	7.18 (4.91, 10.63)	4.97 (3.84, 7.32)	-5.635		<0.001
SCr (umol/L)	66.0 (53.0, 91.0)	88.5 (63.0, 123.3)	64.0 (52.0, 82.5)	-6.286		<0.001
UA (umol/L)	348.0 (270.0, 438.0)	417.0 (304.8, 489.0)	344.0 (278.0, 435.5)	-3.55		<0.001
Cystatin C (mg/L)	1.23 (0.90, 1.77)	1.69 (1.16, 2.33)	1.07 (0.81, 1.44)	-4.983		<0.001
Blood α1-MG (mg/L)	36.3 (30.1, 43.6)	36.3 (33.8, 54.8)	36.3 (27.2, 40.1)	-2.743		0.006
Blood β2-MG (mg/L)	4.61 (3.64, 5.99)	4.88 (4.61, 7.89)	4.61 (2.97, 4.88)	-5.024		<0.001
ESR (mm/hr)	44.0 (25.0, 68.0)	48.0(23.5, 75.0)	43.5 (24.8, 66.0)	-0.989		0.322
CRP (mg/L)	1.90 (0.70, 5.00)	5.00(2.91, 7.80)	1.10 (0.40, 2.20)	-10.734		<0.001
C3 (g/L)	0.46 (0.30, 0.69)	0.43(0.29, 0.66)	0.46 (0.31, 0.70)	-1.104		0.27
C4 (g/L)	0.09(0.04,0.15)	0.08(0.05,0.16)	0.09(0.04,0.15)	-0.245		0.806
Anti-dsDNA, n(%)	182 (50.7)	75 (54.7)	107 (48.2)		1.453 (0.064)	0.228
Anti-Sm, n(%)	90 (25.1)	31 (22.6)	59 (26.6)		0.703 (0.044)	0.402
AnuA, n(%)	139(38.7)	52(38.0)	87(39.2)		0.054(0.012)	0.816
AHA, n(%)	85(23.7)	31(22.6)	54(24.3)		0.135(0.019)	0.713
Anti-rRNP, n(%)	93(25.9)	32(23.4)	61(27.5)		0.749(0.046)	0.387
NLR	3.62(2.34,6.03)	4.70(2.86,7.34)	3.17(2.08,5.04)	-4.586		<0.001
FAR	0.11(0.09,0.16)	0.12(0.10,0.18)	0.11(0.09,0.15)	-2.836		0.005
CLR	1.70(0.61,4.89)	4.80(2.96,9.36)	0.95(0.29,1.92)	-11.443		<0.001
Urine parameters						
Urine WBC, n(%)	137(38.2)	56(40.9)	81(36.5)		0.692(0.044)	0.406
Urine RBC, n(%)	219(61.0)	92(67.2)	127(57.2)		2.471(0.083)	0.116
Cylindruia, n(%)	36(10.0)	16(11.7)	20(9.0)		0.669(0.043)	0.413
Urine α1-MG (ug/L)	16.4(10.8,28.2)	18.3(16.4,45.9)	16.4(8.8,18.3)	-2.743		0.006
Urine β2-MG (mg/L)	0.95(0.34,1.56)	0.95(0.68,2.72)	0.86(0.26,1.13)	-5.024		<0.001
NAG enzyme (U/L)	19.1(12.7,26.6)	16.0(12.7,32.4)	20.1(12.9,24.7)	-0.167		0.868
24h-UTP (mg/24h)	1.72(0.73,3.65)	2.19(1.02,4.06)	1.52(0.69,3.12)	-2.111		0.035
Renal pathological parameters						
Renal biopsy, n(%)	215(59.9)	64(46.7)	151(68.0)		16.004(0.211)	<0.001
Pathological type					2.715(0.112)	0.099
Class Ⅰ, Ⅱ, Ⅴ, n(%)	64(29.8)	14(21.9)	50(33.1)			
Class Ⅲ, Ⅳ, Ⅲ/Ⅳ ± Ⅴ, n(%)	151(70.2)	50(78.1)	101(66.9)			
AI score	3.0(2.0,5.0)	3.0(2.0,7.0)	3.0(2.0,5.0)	-1.68		0.093
Endocapillary hypercellularity	1.0(1.0,2.0)	1.0(0.0,2.0)	1.0(1.0,2.0)	-0.327		0.743
Neutrophils/karyorrhexis	0.0(0.0,1.0)	0.0(0.0,1.0)	0.0(0.0,1.0)	-1.982		0.047
Fibrinoid necrosis	0.0(0.0,0.0)	0.0(0.0,0.0)	0.0(0.0,0.0)	-0.912		0.362
Hyaline deposits	0.0(0.0,0.0)	0.0(0.0,0.0)	0.0(0.0,0.0)	-1.198		0.231
Cellular/fibrocellular crescents	0.0(0.0,0.0)	0.0(0.0,0.0)	0.0(0.0,0.0)	-0.922		0.356
Interstitial inflammation	1.0(1.0,2.0)	1.0(1.0,2.0)	1.0(1.0,2.0)	-2.194		0.028
CI score	3.0(2.0,4.0)	3.0(2.0,4.0)	2.0(2.0,3.0)	-2.906		0.004
Glomerulosclerosis	0.0(0.0,1.0)	0.0(0.0,1.0)	0.0(0.0,1.0)	-1.436		0.151
Fibrous crescents	0.0(0.0,0.0)	0.0(0.0,0.0)	0.0(0.0,0.0)	-0.653		0.514
Tubular atrophy	1.0(1.0,1.0)	1.0(2.0,2.0)	1.0(1.0,2.0)	-2.899		0.004
Interstitial fibrosis	1.0(1.0,2.0)	1.0(1.0,2.0)	1.0(1.0,1.0)	-2.883		0.004
Immune deposition score						
IgA	1.0(1.0,2.0)	1.0(1.0,2.0)	1.0(1.0,2.0)	-0.233		0.824
IgM	2.0(1.0,2.0)	2.0(1.0,2.0)	2.0(1.0,2.0)	-2.028		0.043
IgG	1.0(1.0,2.0)	1.0(1.0,2.0)	1.0(1.0,2.0)	-0.474		0.636
C3	3.0(1.0,3.0)	3.0(2.0,3.0)	2.0(1.0,3.0)	-3.259		0.001
C1q	2.0(1.0,2.0)	2.0(2.0,2.0)	2.0(1.0,2.0)	-0.421		0.674

Correlations between clinical characteristics and CLR/NLR/FAR

**Figure 2:**
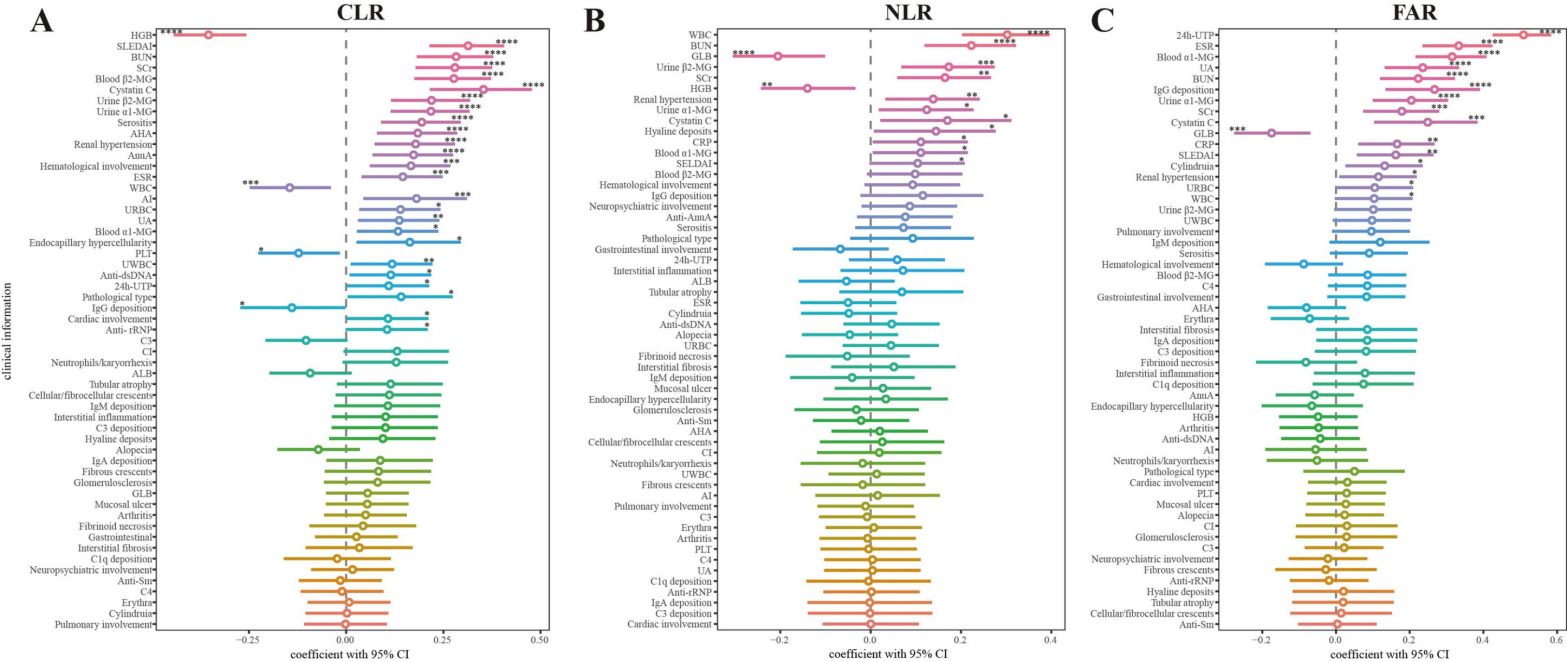
Correlation analysis of (A) CLR, (B) NLR, and (C) FAR with clinical characteristics *p<0.05; **p<0.01; *** p<0.001; ****p<0.0001. SLEDAI - Systemic Lupus Erythematosus Disease Activity Index; WBC - white blood cell; HGB - hemoglobin; PLT - platelets; ALB - albumin; GLB - globulin; BUN - blood urea nitrogen; SCr - serum creatinine; UA - uric acid; MG - macroglobulin; ESR - erythrocyte sedimentation rate; CRP - C-reactive protein; C3 - complement 3; C4 - complement 4; dsDNA - double-stranded DNA; AnuA - anti-nucleosome antibodies; AHA - anti-histone antibodies; Anti-rRNP - anti-ribosomal P-protein autoantibody; UWBC - urine white blood cell; URBC - urine red blood cell; NAG - N-Acetyl-/β-glucosaminidase; UTP - urine total protein; AI - active index; CI - chronic index; IgA - immune globulin A; IgM - immune globulin M; IgG - immune globulin G; C1q - complement 1q

We then analyzed the correlations between clinical characteristics and CLR, NLR, and FAR. As shown in the forest plots (Figure 2), CLR, NLR, and FAR were positively correlated with SLEDAI, BUN, SCr, cystatin C, urine α1-MG, and renal hypertension (all p<0.05). Among the three indices, CLR demonstrated the highest correlation coefficient. We also found specific correlation pairs between CLR, NLR, FAR, and clinical characteristics. For example, CLR was positively correlated with systemic involvement (hematological/cardiac involvement and serositis), autoantibodies (anti-histone (AHA), anti-nucleosome (AnuA), anti-double-stranded DNA (dsDNA), anti-ribosomal P-protein (rRNP)), and pathological parameters (AI score, endocapillary hypercellularity, and pathological type). NLR was positively correlated with hyaline deposits. FAR was positively correlated with cylindruia (all p<0.05). Overall, CLR exhibits the strongest correlations with disease activity and has favorable associations with pathological parameters, which make it a potential biomarker for LN.

Diagnostic accuracy of CLR/NLR/FAR for predicting LN prognosis

**Figure 3:**
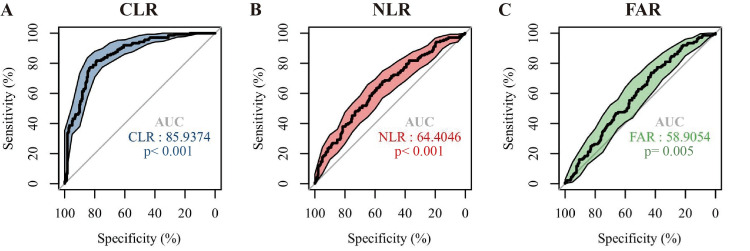
ROC curves of (A) CLR, (B) NLR, and (C) FAR for distinguishing LN with poor prognosis AUC - area under the curve; CLR - C-reactive protein-to-lymphocyte ratio; NLR - neutrophil-to-lymphocyte ratio; FAR - fibrinogen-to-albumin ratio

Receiver operating characteristic (ROC) curves were generated to assess and compare the predictive capability of CLR, NLR, and FAR for LN prognosis. CLR achieved an area under the curve (AUC) of 85.94%, while NLR and FAR had AUCs of 64.41% and 58.91%, respectively (p<0.05; Figure 3). The AUC of CLR was significantly higher than both NLR (z=7.198, p<0.001) and FAR (z=7.681, p<0.001) (Table 3). At optimal cut-off values (CLR: 2.23; NLR: 3.34; FAR: 0.10), CLR showed higher sensitivity (81.8%), specificity (78.8%), and positive predictive value (PPV, 70.4%). and negative predictive value (NPV, 87.5%) for predicting LN prognosis than NLR and FAR (Table 4). These results indicate CLR’s superior performance in predicting poor prognosis of LN compared to NLR and FAR.

**Table 3:** Comparison of AUCs of CLR/NLR/FAR in predicting LN prognosis CLR - C-reactive protein-to-lymphocyte ratio; NLR - neutrophil-to-lymphocyte ratio; FAR - fibrinogen-to-albumin ratio; AUC - area under the ROC curve; CI - Confidence interval

	Z	p-value	AUCdifference	Standard error difference	95%CI lower	95%CI upper
CLR vs. NLR	7.198	< 0.001	0.215	0.222	0.157	0.274
CLR vs. FAR	7.681	<0.001	0.270	0.223	0.201	0.339
NLR vs. FAR	1.308	0.191	0.055	0.245	-0.027	0.137

**Table 4:** Receiver operating characteristics analysis of CLR, NLR, FAR in predicting LN prognosis CLR - C-reactive protein-to-lymphocyte ratio; NLR - neutrophil-to-lymphocyte ratio; FAR - fibrinogen-to-albumin ratio; PPV - positive predictive value; NPV - negative predictive value

Parameters	Cut-off value	Sensitivity (%)	Specificity (%)	PPV (%)	NPV (%)
CLR	2.23	81.8	78.8	70.4	87.5
NLR	3.34	68.0	55.0	48.2	73.5
FAR	0.10	73.7	42.8	43.8	72.6

Kaplan-Meier (K-M) survival analysis in LN patients divided by CLR/NLR/FAR

**Figure 4:**
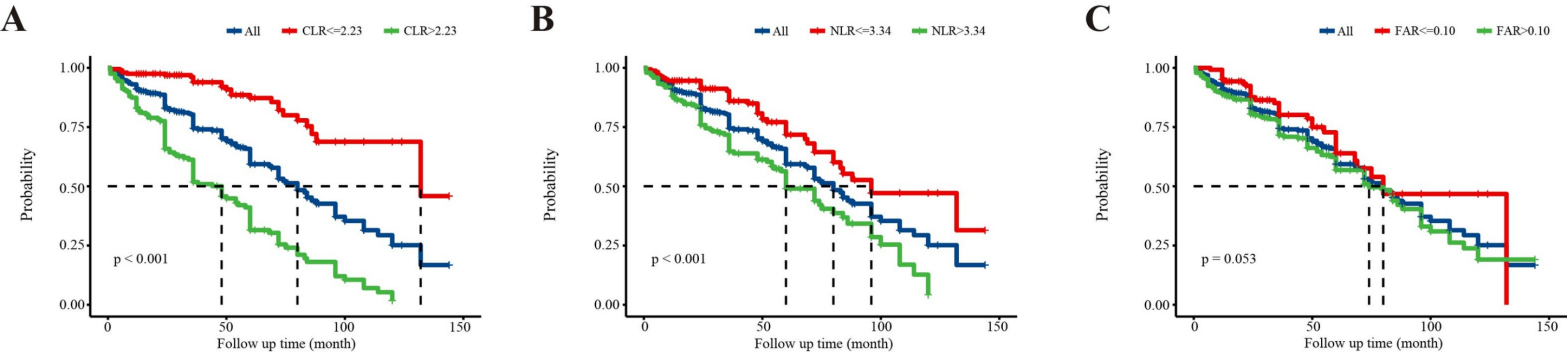
K-M survival analysis calculates cumulative time from enrollment to the composite endpoint in LN patients with high or low levels of (A) CLR, (B) NLR, and (C) FAR CLR - C-reactive protein-to-lymphocyte ratio; NLR - neutrophil-to-lymphocyte ratio; FAR - fibrinogen-to-albumin ratio

To investigate their influence on prognosis, LN patients were categorized by optimal cut-off values of CLR, NLR, and FAR. Kaplan-Meier curves for high and low levels of each index are shown in Figure 4. Patients with higher levels of CLR and NLR had significantly shorter cumulative time to the composite endpoint than those with lower levels (both p<0.05), while no significant difference was observed between groups divided by FAR (p=0.053). These results suggest that high CLR and NLR levels are associated with poor prognosis in LN patients.

LASSO Cox regression identified CLR as an independent predictive indicator for poor prognosis of LN

**Figure 5:**
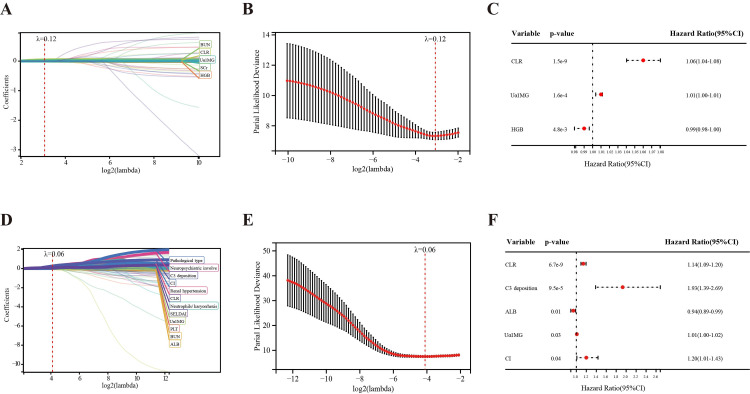
LASSO Cox regression analyzes independent predictors for LN prognosis (A-C) When pathological variables are not included: (A) five variables are selected from 359 LN patients at λ=0.12; (B) λ is confirmed by 10‐fold cross‐validation of LASSO regression using the minimum criteria; (C) CLR, Uα1MG, and HGB are identified as independent predictors by multivariate COX regression. (D-F) When pathological variables are included: (D) twelve variables are selected from 215 LN patients at λ=0.06; (E) λ is confirmed by 10‐fold cross‐validation of LASSO regression using the minimum criteria; (F) CLR, C3 deposition, ALB, Uα1MG, and CI are identified as independent predictors by multivariate COX regression. BUN - blood urea nitrogen; Uα1MG - urine α1 macroglobulin; SCr - serum creatinine; HGB - hemoglobin; CI - chronic index; SLEDAI - Systemic Lupus Erythematosus Disease Activity Index; PLT - platelets; C3 - complement 3; ALB - albumin; LASSO - least absolute shrinkage and selection operator

To screen significant clinical characteristics valuable for predicting LN prognosis, LASSO regression was first conducted to reduce potential collinearity and overfitting among all variables, followed by multivariate Cox regression. When pathological parameters were not included, LASSO regression selected five variables (BUN, CLR, urine α1-MG, SCr, and HGB) from 359 LN patients at λ=0.12 (minimum mean square error; Figure 5A, 5B). Among them, CLR (hazard ratio (HR) =1.06, 95% confidence interval (CI) 1.04-1.08, p<0.001), urine α1-MG (HR=1.01, 95% CI 1.00-1.01, p<0.001) and HGB (HR=0.99, 95% CI 0.98-1.00, p<0.001) were identified as independent predictors by multivariate COX regression (Figure 5C). When pathological parameters were included, LASSO regression selected twelve variables (pathological type, neuropsychiatric involvement, C3 deposition, CI, renal hypertension, CLR, neutrophils/karyorrhexis, SLEDAI, urine α1-MG, platelet (PLT), BUN, albumin (ALB)) from 215 LN patients at λ=0.06 (minimum mean square error; Figure 5D, 5E). Multivariate Cox regression identified CLR (HR=1.14, 95% CI 1.09-1.20, p<0.001), C3 deposition (HR=1.93, 95% CI 1.39-2.69, p<0.001), ALB (HR=0.94, 95% CI 0.89-0.99, p=0.01), urine α1-MG (HR=1.01, 95% CI 1.00-1.02, p=0.03) and CI (HR=1.20, 95% CI 1.01-1.43, p=0.04) as independent predictors (Figure 5F). Among the three composite inflammatory indices, only CLR emerges as an independent prognostic factor for poor prognosis in LN, regardless of whether pathological parameters are considered.

## Discussion

This study is the first to investigate the prognostic value of CLR in LN and compare its efficacy with two established biomarkers, NLR and FAR. Our findings reveal that: 1) CLR, NLR and FAR were significantly elevated in LN patients with poor renal prognosis; 2) CLR was closely associated with renal pathology, and demonstrated the strongest positive correlation with disease activity compared to NLR and FAR; 3) CLR was an independent predictive factor for LN outcome and exhibited the highest prognostic value among the three indices.

Renal failure remains a leading cause of mortality in SLE patients. Early prediction of the long-term renal outcome facilitates optimization of disease management and is vital for LN patients [11]. While renal biopsy provides a reliable evaluation of pathological classification and lesion severity, its invasive nature limits routine application in certain clinical situations. Moreover, repeated renal biopsies are challenging, hindering dynamic monitoring of pathological parameters. Thus, consequently, identifying noninvasive early clinical markers associated with unfavorable renal prognosis is crucial [12].

NLR and FAR are established inflammatory indices that effectively assess disease activity and prognosis in LN [2-4,13-16]. Our analysis supported previous studies, demonstrating higher baseline NLR and FAR levels in LN patients with poor prognosis. Their levels correlated positively with disease activity (SLEDAI), inflammation (CRP), and renal damage indicators (BUN, Scr, cystatin C, urine α1-MG, and renal hypertension). Additionally, ROC curves and K-M survival analysis confirmed their predictive value for LN outcomes. Several studies report close associations between NLR/FAR and renal pathology. For example, NLR levels correlate with AI score, cellular crescents, and tubular atrophy [14]; FAR levels in class Ⅳ LN were higher than those of class Ⅲ LN [4]. However, we only found identified significant positive correlations between NLR and hyaline deposits, and between FAR and IgG deposition. These inconsistencies may result from differences in disease characteristics and sample sizes. Compared with previous research, our study enrolled the largest number of LN patients who underwent renal biopsy. Although NLR and FAR show potential prognostic value, their comparative efficacy and the existence of superior inflammatory indicators remained unexplored.

CLR, integrating CRP and lymphocyte counts, is an emerging biomarker in inflammatory diseases. It independently associates with moderate-to-severe acute pancreatitis incidence [5], predicts survival and postoperative complications in colorectal cancer [6], and differentiates disease severity and ICU admission risk in COVID-19 [7]. These findings suggest a potential role for CLR in LN, another condition characterized by inflammatory responses. However, the relationship between CLR and LN prognosis had not been investigated prior to this study.

We observed positive associations between CLR levels and disease activity (SLEDAI), inflammation (ESR), renal damage indicators (BUN, Scr, cystatin C, blood/urine α1-MG, blood/urine β2-MG, URBC, 24h-UTP, and renal hypertension), systemic involvement (hematological/cardiac involvement and serositis), autoantibodies (AHA, AnuA, anti-dsDNA, and anti-rRNP), and pathological parameters (AI score, endocapillary hypercellularity, and pathological type). The close correlations between CLR and multiple clinicopathological parameters make CLR a robust biomarker for LN patients. Statistically, LASSO Cox regression identified CLR as an independent predictor of LN prognosis, and it achieved the highest AUC compared to NLR and FAR. Kaplan-Meier analysis revealed significant survival differences when stratifying patients by CLR levels. We therefore conclude that CLR possesses superior prognostic value among these three composite indices. Whether or not the pathological parameters are included, the results are consistent, indicating CLR's prognostic value even when a renal biopsy is not performed.

It's known that chronic kidney injury caused by immune cells and inflammation mediators is a hallmark of LN [17]. CRP, an acute-phase protein primarily induced by interleukin-6 (IL-6) during inflammation, typically remains below 60 mg/L in active SLE (except situations of serositis, arthritis, or myositis) but is higher in active versus inactive SLE [18]. Furthermore, CRP levels increase in active LN compared to inactive LN and non-LN patients [19]. Our study found significantly higher median CRP levels in the endpoint group (5.00 mg/L) versus the non-endpoint group (1.10 mg/L), likely reflecting greater inflammation associated with higher SLEDAI scores and proliferative LN prevalence in the endpoint group. Beyond elevated CRP, lymphopenia is frequent in SLE and linked to high disease activity and renal involvement [20,21]. By excluding patients receiving immunosuppressive therapy within the preceding six months, we minimized confounding by treatment-related lymphopenia. Other contributors to lymphopenia include autoantibody production, increased apoptosis/necroptosis, and complement regulatory protein imbalances [22-24]. Consequently, elevated CRP and reduced lymphocytes collectively render high CLR in LN patients with poor prognosis.

Besides CLR, urine α1-MG and HGB were obtained as independent predictive factors for LN prognosis when pathological parameters were not included. Urine α1-MG is a protein with low molecular weight, and can be excreted in large quantities when proximal tubular cells fail in reabsorption. Therefore, increased urine α1-MG means tubular injury in renal diseases [25]. It's reported that urine α1-MG correlates with SLEDAI and is elevated in LN with severe pathological types [26]. In our study, low HGB levels increased the risk of poor renal prognosis. Anemia is more prevalent in LN than non-LN SLE [27]. The occurrence of decreased HGB in LN patients may result from inhibition of renal erythropoietin (EPO) production caused by renal parenchymal infiltration of inflammatory cells and production of anti-EPO antibodies [28]. When pathological parameters were included, the regression identified CLR, C3 deposition, ALB, urine α1-MG, and CI as independent predictors. C3 deposition, characteristic of LN, associates with unfavorable clinical and pathological prognostic factors [29]. ALB, both a nutritional marker and acute-phase protein, decreases in LN due to inflammation-driven capillary leakage and shortened half-life [4]. High CI is a recognized risk factor for ESRD [30]. These previous findings align with our results.

This study evaluated three potential predictors of LN prognosis. The novel inflammatory indicator CLR demonstrated superior ability to identify LN patients with poor prognosis compared to established markers NLR and FAR. There are also some limitations. First, it’s inevitable to have selection bias for this retrospective longitudinal study in a single medical center. Second, only the CLR (at first hospitalization) was analyzed. Their changes during follow-up would provide a more precise assessment, but were unexplored because of missing data. Therefore, prospective multicenter studies are warranted to further validate CLR's predictive value for LN prognosis.

## Conclusions

In conclusion, CLR is a superior prognostic biomarker for renal outcomes in LN, outperforming NLR and FAR. CLR independently predicts poor prognosis, correlates strongly with disease activity and pathology, and demonstrates significantly higher diagnostic accuracy. CLR offers a readily available, non-invasive tool to identify high-risk LN patients early. The utility of CLR in clinical practice needs to be validated by more large-scale studies.

## References

[1] Parikh SV, Almaani S, Brodsky S, Rovin BH (2020). Update on lupus nephritis: core curriculum 2020. Am J Kidney Dis.

[2] Chen X, Lin Z, Chen Y, Lin C (2024). C-reactive protein/lymphocyte ratio as a prognostic biomarker in acute pancreatitis: a cross-sectional study assessing disease severity. Int J Surg.

[3] Okugawa Y, Toiyama Y, Yamamoto A (2020). Lymphocyte-C-reactive protein ratio as promising new marker for predicting surgical and oncological outcomes in colorectal cancer. Ann Surg.

[4] Zhang JN, Gao Y, Wang XT (2022). Lymphocyte-C-reactive protein ratio can differentiate disease severity of COVID-19 patients and serve as an assistant screening tool for hospital and ICU admission. Front Immunol.

[5] Xue L, Shi Y, Zhang J (2022). Correlations of peripheral blood neutrophil-lymphocyte ratio and lymphocyte-monocyte ratio with renal function and prognosis in patients with lupus nephritis. Am J Transl Res.

[6] Zou G, Gao H (2021). The relationship between neutrophil-lymphocyte ratio and early renal fibrosis and renal prognosis in patients with lupus nephritis. Am J Transl Res.

[7] Xu J, Zhang H, Che N, Wang H (2023). FAR in systemic lupus erythematosus: a potential biomarker of disease activity and lupus nephritis. Clin Exp Med.

[8] Hahn BH, McMahon MA, Wilkinson A (2012). American College of Rheumatology guidelines for screening, treatment, and management of lupus nephritis. Arthritis Care Res (Hoboken).

[9] Levey AS, Gansevoort RT, Coresh J (2020). Change in albuminuria and GFR as end points for clinical trials in early stages of CKD: a scientific workshop sponsored by the National Kidney Foundation in collaboration with the US Food and Drug Administration and European Medicines Agency. Am J Kidney Dis.

[10] Arends S, Grootscholten C, Derksen RH (2012). Long-term follow-up of a randomised controlled trial of azathioprine/methylprednisolone versus cyclophosphamide in patients with proliferative lupus nephritis. Ann Rheum Dis.

[11] Gasparotto M, Gatto M, Binda V, Doria A, Moroni G (2020). Lupus nephritis: clinical presentations and outcomes in the 21st century. Rheumatology (Oxford).

[12] Parodis I, Tamirou F, Houssiau FA (2020). Prediction of prognosis and renal outcome in lupus nephritis. Lupus Sci Med.

[13] Ayna AB, Ermurat S, Coşkun BN, Harman H, Pehlivan Y (2017). Neutrophil to lymphocyte ratio and mean platelet volume as inflammatory indicators in systemic lupus erythematosus nephritis. Arch Rheumatol.

[14] Han Q, Liang P, Li J (2024). The ratio of neutrophil to lymphocyte as a potential marker of clinicopathological activity for lupus nephritis. Int Urol Nephrol.

[15] Chen Y, Wu X, Chen X (2023). Correlations of baseline neutrophil-lymphocyte ratio with prognosis of patients with lupus nephritis: A single-center experience. Rheumatol Immunol Res.

[16] Tang D, Tang Q, Zhang L, Wang H (2022). High neutrophil-lymphocyte ratio predicts serious renal insufficiency in patients with Lupus Nephritis. Iran J Immunol.

[17] Maria NI, Davidson A (2020). Protecting the kidney in systemic lupus erythematosus: from diagnosis to therapy. Nat Rev Rheumatol.

[18] Aringer M (2020). Inflammatory markers in systemic lupus erythematosus. J Autoimmun.

[19] Bona N, Pezzarini E, Balbi B (2020). Oxidative stress, inflammation and disease activity biomarkers in lupus nephropathy. Lupus.

[20] Kandane-Rathnayake R, Louthrenoo W, Golder V (2021). Independent associations of lymphopenia and neutropenia in patients with systemic lupus erythematosus: a longitudinal, multinational study. Rheumatology (Oxford).

[21] Lioulios G, Mitsoglou Z, Fylaktou A (2022). Exhausted but not senescent T lymphocytes predominate in lupus nephritis patients. Int J Mol Sci.

[22] Noguchi M, Iwamori M, Hirano T, Kobayashi S, Hashimoto H, Hirose S, Nagai Y (1992). Autoantibodies to T and B cell lines detected in serum samples from patients with systemic lupus erythematosus with lymphopenia and hypocomplementaemia. Ann Rheum Dis.

[23] Katsuyama T, Martin-Delgado IJ, Krishfield SM, Kyttaris VC, Moulton VR (2020). Splicing factor SRSF1 controls T cell homeostasis and its decreased levels are linked to lymphopenia in systemic lupus erythematosus. Rheumatology (Oxford).

[24] Fan H, Liu F, Dong G (2014). Activation-induced necroptosis contributes to B-cell lymphopenia in active systemic lupus erythematosus. Cell Death Dis.

[25] Bazzi C, Petrini C, Rizza V, Arrigo G, Beltrame A, Pisano L, D'Amico G (2001). Urinary excretion of IgG and alpha(1)-microglobulin predicts clinical course better than extent of proteinuria in membranous nephropathy. Am J Kidney Dis.

[26] Ding J, Zheng Z, Li X, Feng Y, Leng N, Wu Z, Zhu P (2017). Urinary albumin levels are independently associated with renal lesion severity in patients with lupus nephritis and little or no proteinuria. Med Sci Monit.

[27] Sharma M, Das HJ, Doley PK, Mahanta PJ (2019). Clinical and histopathological profile of lupus nephritis and response to treatment with cyclophosphamide: a single center study. Saudi J Kidney Dis Transpl.

[28] Ardalan MR (2013). Anemia in lupus nephritis; etiological profile. J Renal Inj Prev.

[29] Koopman JJ, Rennke HG, Leatherwood C (2020). Renal deposits of complement factors as predictors of end-stage renal disease and death in patients with lupus nephritis. Rheumatology (Oxford).

[30] Zoshima T, Hara S, Suzuki K (2024). Long-term outcomes of lupus nephritis with low-level proteinuria: a multicentre, retrospective study. Rheumatology (Oxford).

